# Descriptive Epidemiology of Male Breast Cancer in Osaka, Japan

**DOI:** 10.2188/jea.11.1

**Published:** 2007-11-30

**Authors:** Naoko Tajima, Hideaki Tsukuma, Akira Oshima

**Affiliations:** 1 Community Health Nursing, Osaka Prefectural College of Nursing.; 2 Department of Cancer Control and Statistics, Osaka Medical Center for Cancer and Cardiovascular Diseases.

**Keywords:** male breast cancer, incidence, survival, population-based cancer registry

## Abstract

Male breast cancer is rare. The total number of incidence in Osaka for the period of 1966-95 was 182. Male-to-female ratio for breast cancer incidence was 1:164 in Osaka during this period. Mean age of the male breast cancer incidence was 63.3. The numbers of incidence and the crude incidence rates for male breast cancer have increased during the last 3 decades, while the age-standardized rates have remained constant. The age-specific incidence rates for males showed a gradual increase with age, while those for females showed a steep increase beginning at twenty years of age and a peak around 45-49 or 50-54 years old. The age-standardized incidence rates of male breast cancer were lower in Japan than in European countries and North America, as were those of female breast cancer. Distributions of the histological type and the extent of disease were not significantly different between males and females. Relative 5-year survival for the male breast cancer was, however, lower than that for the female, especially in the “regional” stage and “distant” stage. Further studies on the sex-difference in survival will be mandatory based on high-quality hospital cancer registries’ data, which provide detailed information on the clinical stage and treatment.

## INTRODUCTION

Male breast cancer is rare and little is known about the incidence and prognosis of this disease in Japan. Incidence and survival of male breast cancer in Osaka, Japan will be presented, described and compared with data on female breast cancer in Osaka and with data in other countries populations.

## MATERIALS AND METHODS

The recent 3 decades’ data of the Osaka Cancer Registry (OCR) was employed. The OCR, one of the largest population-based cancer registries in Japan, has been in operation since December 1962 and covers the Osaka population of 8.8 million (1995 census)^1^^)^. Since the quality of data was not very good in the early years of the registry, incident analysis was based on the period of 1966-95. For survival analysis, those data on the cases diagnosed in 1975-91 and that lived in Osaka Prefecture, excluding Osaka City, was used since these cases were actively followed up and their vital statuses at 5 years after the diagnosis were confirmed almost perfectly. Those cases registered by death certificates only, however, were excluded from the survival analysis. Cumulative 5-year survival was estimated by using the Kaplan-Meier’s method. Five-years’ relative survival was calculated by dividing the cumulative 5-year survival by corresponding 5-year expected survival, which was obtained based on the cohort survival tables prepared by the National Cancer Center, Tokyo, Japan^2^^)^. In this study three categories of stage, i.e., “localized” (confined to the breast tissue), “regional” (spread to regional lymph-nodes and/or spread to immediately adjacent tissues), and “distant” (metastasis to distant organs) were used in the analysis of stage distribution and survival by stage. This classification of stage at diagnosis is used not only in the OCR but also in most population -based cancer registries^3^^)^.

International comparisons of cancer incidence were based on the data from Cancer Incidence in Five Continents (CI5) Volume VII^4^^)^, which includes basically 1988-92’s cancer incidence data for 183 populations in 50 countries from 150 cancer registries.

The chi-square and the Mann-Whitney tests were used for comparisons of the histological type and the extent of disease distributions between males and females, respectively. Greenwood’s formula was employed for testing statistical differences in 5-year survival between males and females. P-values less than 0.05 were judged as statistically significant. Statistical package software, STATA^5^^)^ was used for statistical analysis.

## RESULTS

### Incidence and its time-trends

**Figure 1.**
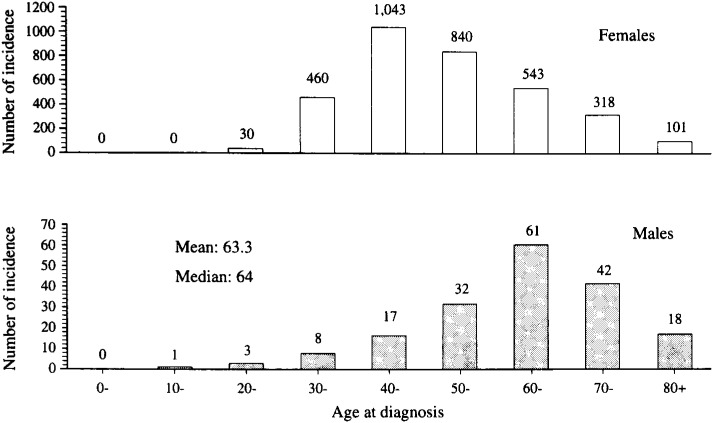
Age-specific incident cases of male breast cancer in Osaka during the period of 1966-95, together with the females’ data for the period of 1981-83.

The total number of male breast cancer incidence was 182 in Osaka during 1966-95. In Table 1, the number of incidence, crude incidence rates (per 100,000 population), age-standardized rates (standardized to World population), proportion of cases with death certificate only (DCO%), and percentage of histologically verified cases (HV/R%) are presented in ten-year intervals, together with the females’ data for the period of 1981-83, the middle of the total study period. The numbers of incidence and the crude incidence rates have increased in the last decades, although the age-standardized rates remained constant. During the same period, the age-standardized incidence rates for female breast cancer have increased more than 100% (11.8→23.7). DCO% and HV/R% for breast cancer in Osaka have been almost constant during this period for males (about 10% and 90 %, respectively), while corresponding indices for females decreased from 7.6% to 3.1% and increased from 76.3% to 92.1%^1^^)^. Figure 1 shows the age-specific incident cases of male breast cancer in Osaka during the period of 1966-95, together with the females’ data for the period of 1981-83. The mean age of those cases was 63.3 years old and the median age was 64 years old.

**Table 1.** Male breast cancer in Osaka, 1966-95 and data for female’s, 1981-83.

	Males				Females
	
	Periods			Total	Periods
	
	1966-75	1976-85	1986-95		1981-83
	
No. of incidence	47	50	85	182	3,338
Crude incidence rate	0.12	0.12	0.20	0.15	25.79
Age-standardized rate	0.22	0.19	0.22	0.21	21.3
DCO%	8.5	12.0	9.4	9.9	3.8
HV/R%	74.4	88.6	92.2	86.6	91.9

DCO%: Proportion of cases registered by death certificates only

HV/R%: Proportion of cases verified histologicaly

### Comparison of incidence rates among males and females, Osaka and US SEER/Other populations

**Figure 2.**
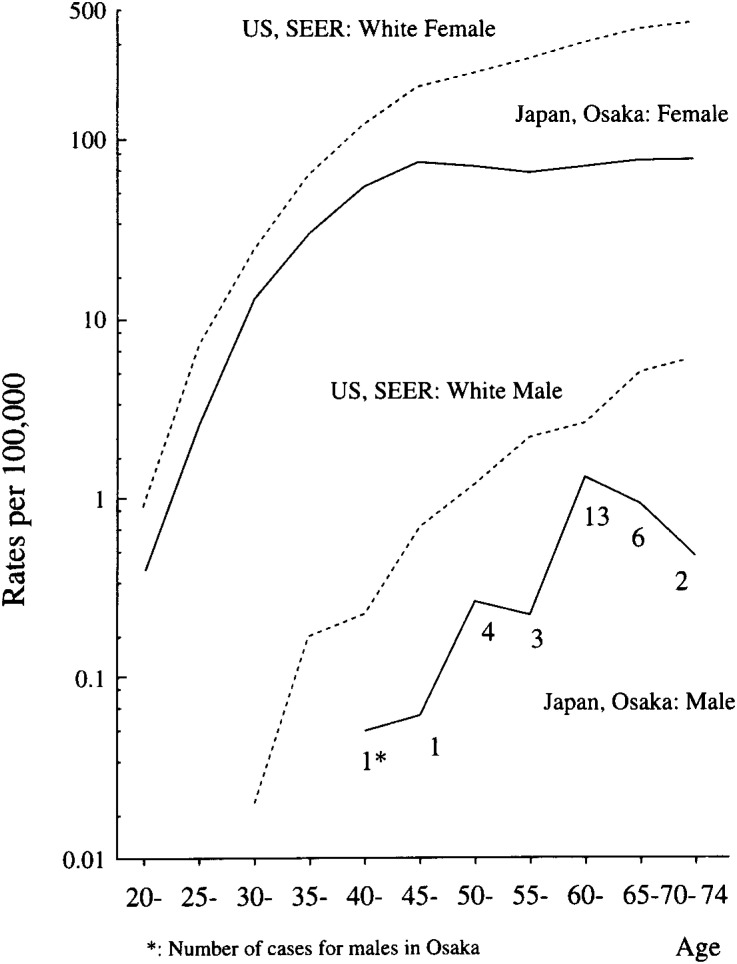
Age and sex-specific incidence rates of breast cancer in Osaka and the US whites in 1988-92.

Figure 2 indicates the age and sex-specific incidence rates of breast cancer in Osaka and the US whites population in 1988-92^4^^)^. Males in Osaka showed a gradual increase with age starting from 40-44 years old, while females in Osaka showed a steep increase starting at twenty years of age. These differences of incident increase starting ages and its slope for the age-specific incidence rates between males and females were also observed for the US whites. The age-specific incidence rates for Japanese females peaked around 45-49 or 50-54 years old and showed a slight decrease or plateau thereafter. These characteristics of the age-specific incidence curve of breast cancer for Japanese females were explained by the so-called birth-cohort effect. For Japanese males, however, the age-specific incidence rates were not appreciably different by birth-cohort (data not shown).

**Figure 3.**
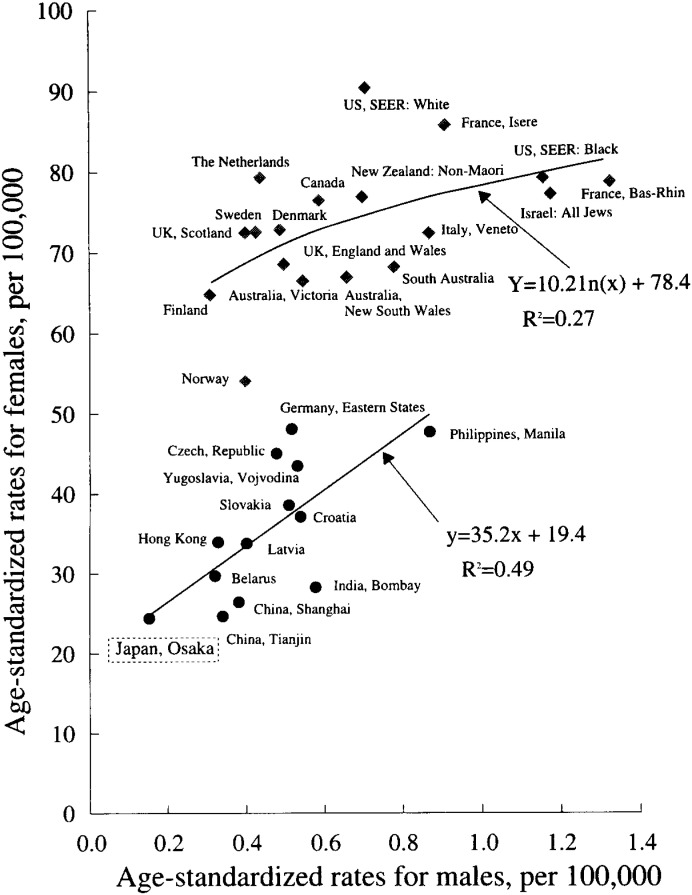
International correlations of the age-standardized incidence rates of breast cancer between males and females.

In Figure 3, scatter plots are presented, showing international correlation of the age-standardized incidence rates of breast cancer between males and females. Thirty one cancer registries’ data were selected from CI5 Volume VII^4^^)^ so that at least 30 male breast cancer cases could be included for plotting each registry/country data. Generally the age-standardized rates for males correlated well with those for females internationally (Pearson product-moment coefficient of correlation=0.58, P=0.0007). Two groups of populations, however, seem to be distinguishable. The first is northern, western and southern European countries (Norway, Finland, Sweden, Denmark, UK, The Netherlands, Italy, France), North America, New Zealand and Australia. The second is eastern European countries (Belarus, Latvia, Croatia, Slovakia, Yugoslavia, Czech Republic, Eastern States of Germany) and Asian countries (Osaka, China, India, Hong Kong, Philippines, Manila). The first group of populations showed much higher incidence rates of female breast cancer than the second one, and the females’ rates in the first group seemed to reach a plateau for populations with highest male breast cancer risk. In the second group, the females’ rates seemed to linearly correlate well with the males’ rates.

### Distribution of histological types

In Table 2, distribution of histological types^6^^)^ of male breast cancer cases in Osaka is presented and compared with data on 2,508 female cases diagnosed in 1981-83, the middle of the total study period. Among the 182 male breast cancer cases, detail information on histology was obtained from 97 patients. In general, distribution of the histological types was not significantly different between males and females (Chi-square=2.997 d.f.=5, P=0.7). However, proportion of the special type in males was higher than that in females (26.8% vs. 9.2%), while proportion of the scirrhous carcinoma in males was lower than that in females (11.3% vs. 15.4%).

**Table 2.** Histological type of male breast cancer in Osaka, 1966-95 and data for female’s 1981-83.

Histological type	Males		Females	
	
	Diagnosed in 1966-95		Diagnosed in 1981-83	
	
	No. of cases	%	No. of cases	%
1. Noninvasive carcinoma				
Noninvasive ductal carcinoma	2	2.1	19	0.8
2. Invasive carcinoma				
(1) Invasive ductal caricinoma				
Papillotubular carcinoma	17	17.5	576	23.0
Solid-tubular carcinoma	41	42.3	1,286	51.3
Scirrhous carcinoma	11	11.3	385	15.4
(2) Special type	26	26.8	229	9.2
3. Paget’s disease	0	0.0	13	0.5

Total	97	100.0	2,508	100.0

### Extent of disease and its time-trends

In Table 3, time-trends of the extent of disease (clinical stage) are presented in ten-year periods for males, together with data for females in 1981-83. Proportions of the localized stage and the distant stage have increased in the last two decades, while percentage of the regional stage has decreased. In the total study period, distribution of the extent of disease among the male breast cancer cases did not differ significantly from that among the females (Mann-Whitney test, P=0.38), although percentage of the unknown extent of disease was higher among males than females (26.4% vs. 5.3%).

**Table 3.** Distribution of the extent of disease and its time trends -Male and female breast cancer in Osaka.

Extent of disease	Males								Females	
	
	1966-75		1976-85		1986-95		Total		1981-83	
				
	No. of cases	%	No. of cases	%	No. of cases	%	No. of cases	%	No. of cases	%
Localized (including in situ)	10	33.3	20	54.1	36	53.7	66	49.3	1,437	51.5
Regional	19	63.3	13	35.1	24	35.8	56	41.8	1,207	42.3
Distant	1	3.3	4	10.8	7	10.4	12	9.0	145	5.2

Unknown	(17)	-	(13)	-	(18)	-	(48)	-	(157)	-

(Number of “in situ”: 1 for males, 24 for females)

### Relative 5-year survival and its comparison between males and females

In Table 4, relative 5-year survival for the male breast cancer was presented according to the extent of disease, together with the females’ data for the period of 1981-83. In total, relative 5-year survival was lower in males than in females (57.8% vs.80.1%, P=0.004). Stage-specific survivals were also consistently lower in males than in females. Statistically significant difference in the survival between males and females was observed for the regional (36.7% vs. 73.3%, P=0.003).

**Table 4.** Relative 5-year survival according to the extent of disease -Male breast cancer in Osaka, 1975-91 and data for female’s, 1981-83.

Extent of disease	Females			Females		
	
	Diagnosed in 1975-91			Diagnosed in 1981-83		
	
	No. of cases	( % )	RSR%	No. of cases	( % )	RSR%
Localized	27	( 45.0 )	87.3	885	( 46.3 )	96.5
Regional	20	( 33.3 )	36.7	748	( 39.1 )	73.3
Distant	4	( 6.7 )	0.0	87	( 4.6 )	22.4
Unknown	9	( 15.0 )	41.3	191	( 10.0 )	56.7

Total	60	( 100.0 )	57.8	1,191	( 100.0 )	80.1

RSR: Relative 5-year survival

## DISCUSSION

As far as the authors know, this is the first report on a population-based study on the incidence and survival of male breast cancer in Japan. Male breast cancer incidence in Osaka, Japan was suggested to belong to the lowest risk group in the world, and this coincided with females’ lower risk for breast cancer in Osaka.

Although the incidence numbers and the crude incidence rates of male breast cancer in Osaka have increased, the age-standardized rates have remained constant during the last 3 decades (Table 1). This contrasts with more than 100% increase in the female breast cancer risk in Osaka. Since DCO% for female breast cancer in Osaka has decreased, the increase in the age-standardized incidence rates might be partly affected by improved reporting to cancer registry. This discrepancy in the time-trends of breast cancer between males and females, however, might be a reflection of different etiologies of breast cancer between the two sexes. Earlier ages at menarche and later ages at the first birth among Japanese women would be major reasons for the increase of female breast cancer in Japan^7^^,^^8^^)^.

Differences in the age-specific incidence curve between males and females also suggested different determinants of breast cancer risk between males and females (Figure 2). The age distribution of male breast cancer in Osaka was almost the same (Mean age: 60 years) as the other papers from the US and Iceland^9^^,^^10^^,^^11^^,^^12^^)^ (Figure 1). An earlier and steeper increase of the age-specific incidence rates of female breast cancer could be explained by its strong estrogen dependence and breast tissue immaturity before the first delivery; while male breast cancer would be barely concerned with adipose tissue converted estrogens from adrenal androgens and accumulation of environmental risk factors^8^^)^.

The observed significant positive international correlation between males’ and females’ breast cancer incidences may suggest the roles of common etiological factors for breast cancer. Although there is no evidence supporting it, the so-called calorie rich dietary habits would be the most likely explanation for the international correlation. Besides the calorie rich dietary habits, reproductive histories of women i.e., later ages at the first delivery or no childbirth would have caused additional risk for breast cancer in the first group of countries and populations (northern, western and southern European countries, North America, Oceania), showing the highest female breast cancer incidence in Figure 3^13^^,^^14^^,^^15^^)^.

As for the stage and survival of male breast cancer in Osaka, the proportion of the localized stage has increased in the last 3 decades (Table 3). However, relative 5-year survival for males was still lower than that for females (Table 4), even though their survival (57.8%) was almost comparable to Nordic Countries’ data (Denmark: 53.8%, Finland: 47.7%, Norway: 66.3%, Sweden: 63.8%)^16^^)^. The lower survival could not be explained only by the delay of diagnosis for male breast cancer in Osaka, since their stage-specific survivals were also lower than those for females^17^^)^. These results on the sex-difference in the survival were inconsistent with reports from the US and Iceland^9^^,^^10^^,^^15^^,^^16^^)^, where males’ poor survival could be explained by more advanced stages at diagnosis. Percentage of scirrhous carcinoma in males was rather lower than in females in this study (Table 2).

Finally the study limitations and the future research areas for male breast cancer should be discussed. First of all, completeness of the registration for male breast cancer incidence might not be satisfactory when compared with data from North America and Europe. Ajiki et al.^25^^)^ proposed a formula to estimate completeness of cancer registration based on DCN%^26^^)^ (proportion of the cases first notified via death certificate) and I/D (incidence to mortality) ratio, i.e., the degree of completeness %= (1-DCN% × I/D ratio) / (1-DCN%). Tentatively, DCN was defined as sum of DCO plus cases not reported but with supportive clinical information of cancer obtained through the tracing back survey, as suggested by Ajiki et al. Proportion of DCN of breast cancer in Osaka for 1993-95 was calculated as 15.4% for males and 9.4% for females, while the I/D ratio was 2.0 and 3.3, respectively. The degree of completeness was thus estimated as 81.8% for males and 76.6% for females. Although we could not estimate the degree of completeness during the whole study period, the incidence of male breast cancer in Osaka should be regarded as underestimated.

Secondly, missing data on the histological type and the extent of disease was rather high in this study. The number of breast cancer incidence was still very small. Thus the possibilities of misclassifications and random variations should be taken into consideration. Finally, the extent of disease used in this study might not be adequate for precise evaluations of clinical stage and stage-specific survival. Further studies, especially on the sex-difference in the survival, will be mandatory based on high-quality hospital-based site-specific cancer registries, which will provide us detailed information on the clinical stage and treatment.
